# The Hospital Officer's Bookshelf

**Published:** 1913-02-01

**Authors:** 


### THE HOSPITAL OFFICER'S BOOKSHELF.

Medical Diseases of Children. By T. R. C. Whipham, M.A., M.D. (Oxon.). Illustrated. (London: University of London Press, Hodder and Stoughton, and Henry Frowde. 1913. Pp. 417. Price 10s. 6d.)

This text-book may perhaps best be described as a convenient compilation, since it appears to be lacking those characteristics of individuality which mark the writings of all who draw upon their own personal experience. This manual has some good points, but it is difficult to see wherein lies its advantages over other text-books of a similar scope. As a text-book for students it may serve a useful purpose, for the author does not waste words, but tells a plain unvarnished tale. The ground is covered in a somewhat skimpy fashion and the "accepted teaching" served up in a more or less readable manner. But, unfortunately, this book can scarcely be described as really helpful to practitioners. The latter portion of the volume gives one a more favourable impression than the first part, where, especially in the chapters on the specific infectious fevers, there are several weak spots. The mortality of children under five from scarlet fever is nothing like "20 to 30 per cent."; nor should the dosage of diphtheria antitoxin be graded according to the age of the child; and it is not the mild cases of chicken-pox which are readily diagnosed, but these are the very ones which present most difficulty in a modern smallpox outbreak. Other points of variance with modern methods occur in this section, which requires a good deal of revision. The section on nervous diseases appears more congenial to the author, and here he is distinctly aided by the numerous good photographs illustrating the conditions.

Pye's Surgical Handicraft: A Manual of Minor Surgery. Edited by W. H. Clayton Greene, F.R.C.S. Sixth Edition. (Bristol: John Wright and Sons, Ltd. 1912. Price 12s. 6d. net.)

To successive terms of students "Pye" has become a surgical text-book of unequalled excellence because of its conciseness, its thorough grasp of the details of minor surgery and those manipulations which are familiar—or are supposed to be familiar—to the dresser, and its clear, terse, and generally accurate directions. What it teaches cannot, after all, be well learned from books; these details of minor surgery and surgical handicraft must be mastered in the out-patient department or in the surgical ward under the guidance and supervision of an experienced teacher. But such books are always helpful, though they do not claim to make the experienced teacher a superfluity. They impress certain essential points on the memory of the student; they are helpful, moreover, to the qualified man who, through no fault of his own but merely by reason of the fact that he has not had sufficient practice since passing his final, is in danger of becoming "stale" on all these nice little points of technique. No one, therefore, can honestly say that "Pye" and its rivals do not, to use a common term of expression, supply "a much needed want." This, the sixth edition of the familiar text-book, has been almost rewritten by Mr. Clayton Greene with the assistance of several teachers who have devoted themselves to special subjects. Thus Mr. Paton deals with the minor surgery of the eye; Mr. Carson with manipulations on the nose and throat, with common diseases of the larynx, and with diseases of the ear. Mr. Norman Bennett supplies a very useful and practical article on dental matters; the hints on extraction are of great value to the student who has not had an opportunity of learning the technique as dental dresser. Dr. Willcox contributes a chapter on the treatment of cases of poisoning, and another on urine testing. Dr. Blumfeld writes on anæsthesia, and Mr. Simmons contributes an interesting article on $x$-rays and the interpretation of skiagraphic plates. All these special articles, while they make the book somewhat more extended and less concise than was the older edition, contribute largely to its final usefulness, though in its present shape "Pye" cannot be considered as a true pocket-book. The information contained in the various chapters has been brought up to date; it is everywhere clearly stated, and the directions given to the student are concise and accurate. Some valuable new illustrations have been added, so that in this direction, too, the manual is commendable, though we have always held that blocks are of comparatively little use in a book of this kind since the student needs to have seen and mentally registered the various appliances and methods which he will be called upon to use. Clinical hints are scattered broadcast through the volume. We have no intention of going into detail. A critical teacher will doubtless find points here and there on which he is not in thorough agreement with the writers, but these are matters largely of opinion, and do not affect the book as a whole. In its present form the sixth edition of "Pye" is a manual which we can wholeheartedly recommend to the senior student who is starting his ward work; he will find it one of the most useful and informative books in the publishers' catalogues, and the investment of its listed value in purchasing it will be fully warranted by the benefit he will derive from its perusal. Not less valuable will the book be to the general practitioner, who will find it a useful reference manual to be appealed to in cases of doubt.

